# Missed opportunities for earlier HIV testing and diagnosis at the health facilities of Dessie town, North East Ethiopia

**DOI:** 10.1186/1471-2458-10-362

**Published:** 2010-06-23

**Authors:** Netsanet W Fetene, Amsalu D Feleke

**Affiliations:** 1 EngenderHealth, Amhara Regional Office, Dessie, Ethiopia; 2 Department of School of Public Health, College of Medicine and Health Sciences, University of Gondar, Gondar, Ethiopia

## Abstract

**Background:**

For patients in all health-care settings HIV screening is recommended after the patient is notified that testing will be performed unless the patient declines. The nation's physicians and other health care providers should assume a much more active role in promoting HIV testing. The aim of this study was to investigate the extent to which missed opportunities for earlier HIV testing and diagnosis occur in the health facilities of north east Ethiopia.

**Methods:**

A confidential client exit interview and medical record review was made on 427 clients who attended health facilities of Dessie town between November-December 2008. Data collection was done by counselors trained on Provider Initiated Counseling and Testing (PICT) and data collection tool included demographics, reason for visit to health facilities, HIV test initiation by service providers, clients self risk perception, clients willingness and acceptance of HIV test, HIV test result and review of client medical records.

**Results:**

Among 427 clients, missed opportunities for HIV testing were found in 76.1% (325) of clients. HIV test initiation was made by data collecting counselors during interview period and 80.0% (260) of clients not initiated by service providers were found to be willing to have HIV test. Large number, 43.0% (112), of the willing clients actually tested for HIV. Of the tested clients, 13.4% (15) were found to be HIV positive. Most, 60% (9), of HIV positive clients who lost the opportunities of diagnosis felt themselves as having no risk for HIV infection. Missed opportunities for HIV diagnosis of 51.7% (15), overall HIV test acceptance rate of 36.5% (154) and positivity rate of 6.9% (29) were found.

**Conclusions:**

The missed opportunities for earlier HIV test and diagnosis of patients attending health facilities were found to be high and frequent. Testing only clients with HIV risk misses large number of HIV positive patients. Asking clients' willingness for HIV testing should be conducted by all service providers irrespective of the clients' risk behaviors for HIV infection or the type of services they need.

## Background

AIDS is the gravest global pandemic of our time. It has already claimed over 20 million lives, with another 39 million individuals currently estimated to be living with HIV worldwide, and millions more becoming newly infected each year[1]. Sub-Saharan Africa continues to bear the brunt of the global epidemic. Two thirds (63%) of all adults and children with HIV globally live in sub-Saharan Africa[2]. The overall HIV prevalence estimate for Ethiopia in 2007 using single point estimate was 2.1% and is in increasing trend[3]. The HIV incidence since 2005 was 0.26% and is expected to remain stable until 2010[4].

More than 2 decades after AIDS was first described, patients continue to present for initial HIV-related medical care years after acquiring the virus. Although delays do occur between HIV testing and linking to care, the greatest delay occurs between initial infection and HIV testing [5]. As we begin the third decade of the epidemic, we still have far to go. In some respects, as much as we have learned about HIV, we have not succeeded on some very basic fronts; most important, in the core public health functions of ensuring that people learn their HIV status, reduce their risky behaviors, and have access to new treatments that might prolong life [6].

Voluntary counseling and testing for human immune deficiency virus (HIV) has been carried out in many places with excellent results; it is cost-effective, and a gateway to most HIV related services including provision of antiretroviral drugs [7]. Yet, the current reach of HIV testing services remains poor. In low and middle income countries only 10 per cent of those who need voluntary counseling and testing have access to it because they may have been exposed to HIV infection [8]. As a result, in most sub-Saharan African countries, many people still do not know their HIV status [7].

Over a decade has passed since an early call to action about HIV testing was prominently stated, "The nation's physicians and other health care providers should assume a much more active role in promoting HIV testing"[5]. PIHCT (Provider Initiated HIV Counseling and Testing), a recently introduced program focuses on suspected clients and high risk areas of health care services. Clinic protocol requires doctors or health advisers seeing new patients to ask about and record risk factors for HIV infection and over information about HIV testing to all regardless of risk [9]. Despite public health policies promoting more aggressive HIV testing, physicians generally view the HIV test as a diagnostic test rather than as a screening test [10]. The value of HIV testing goes beyond enabling medical care for the infected individual. Some studies have demonstrated that knowledge of HIV serostatus, particularly when positive, decreases behavior that can result in HIV transmission [5]. Most people who learn they are HIV-positive are motivated to adopt safer behaviors so that they do not infect others [11]. In addition, approximately 2.9 million lives have been saved because of access to antiretroviral therapy and an estimated 11.7 million life years were added globally between 1996 and 2008 as a result of antiretroviral therapy [12].

A study conducted at a South Bronx from 1992-1994 to identify the extent of missed opportunities for offering HIV counseling and voluntary testing (CT) to residents of a community with a high prevalence of HIV infection, showed that of the 807 patients enrolled 411 (51%) had never been offered HIV CT previously[13].

From 10-year retrospective chart review of patients seen at an HIV intake clinic between January 1994 and June 2001 at Boston Medical center, triggers were found in 50% of patients. This study showed missed opportunities for addressing HIV testing remains unacceptably high when patients seek medical care in the period before their HIV diagnosis[5]. Moreover, in settings serving clients at increased behavioral and clinical risk for HIV infection, targeting HIV testing based on reported risk factors will miss many HIV-infected clients[14].

Data from the US Centers for Disease Control and Prevention (CDC) were released showing that in 25 states with HIV reporting, 41% of people infected with HIV learned their status long after infection, either at the same time as or within 1 year of an AIDS diagnosis [6]. In the United States 30% of infected individuals are unaware of their diagnosis; as many as 275,000 people are infected with HIV but do not know it [5].

The revised CDC recommendation for HIV testing suggests that for patients in all health-care settings HIV screening is recommended after the patient is notified that testing will be performed unless the patient declines (opt-out screening). Persons at high risk for HIV infection should be screened for HIV at least annually[15]. Recent data indicate that routine HIV testing at least once may be cost effective, even in areas with sero prevalence less than 1%[16].

Botswana began routine, noncompulsory (i.e., "opt-out") HIV screening in prenatal and other health-care settings [17]. In Ethiopia, the National TB and HIV guideline recommends HIV counseling and testing as a routine care for TB patients [18]. As the result, since the end of 2005, more than 330 health facilities offer provider initiated counseling and testing at TB clinics [19]. The Ethiopian HIV guideline encourages HIV testing and counseling to patients who do not exhibit obvious HIV-related symptoms and signs. Such HIV testing and counseling is recommended by the health care provider as part of a package of services provided to all patients during all clinical interactions in the facility [20].

It is recommended by CDC that all persons aged 13-64, regardless of risk, should receive routine, voluntary screening for HIV in all healthcare settings in which the prevalence of undiagnosed HIV infection is at least 0.1% [21]. In Ethiopia, persons 15 years and above are considered mature enough to give informed consent for themselves. However, children aged 13-15, who are married, pregnant, commercial sex workers, street children, heads of families, or sexually active are regarded as "mature minors" who can consent to HIV testing [20].

Therefore, it would become missed opportunity for most clients who were at risk or already infected but failed to be told by the health worker to have the test at the right time. Such problems considerably hamper HIV prevention and treatment intervention at individual and at community level. The extent to which missed opportunities for HIV testing and diagnosis occur in medical evaluations prior to one's HIV diagnosis is not known.

The general objective of the study was to investigate the extent to which missed opportunities for earlier HIV testing and diagnosis occur in the health facilities of Dessie town.

## Methods

A cross-sectional quantitative study design on selected health facilities was applied. The method used systematically randomized clients exit interview and reviewing of clients' chart.

The study was conducted in Dessie town from Nov 30/2008-December 21/2008. Dessie is an urban zonal town located in south Wollo zone, Amhara National Regional State, Ethiopia. In the town, there are three health centers and one hospital owned by the government, two non-government clinics and privately owned three hospitals and five higher clinics.

The total population of Dessie town was 198,801. The study unit of this research involved clients attending four government health facilities (one hospital and three health centers) and the two NGO clinics; Dessie Marie Stops International (MSI) clinic and Family Guidance Association of Ethiopia (FGAE) clinics. The private health facilities were deliberately not included because of the different socio-economic characteristic of clients coming to these facilities from government and NGO clinics and due to the high cost associated with HIV test which possibly affects the test acceptability rate.

A total of 427 clients participated in the exit interview part of the study. Since there was no previous study done in our set-ups on the missed opportunities for both HIV testing and diagnosis, in order to get the maximum sample size, 50% of clients coming to the facility were considered to have missed opportunities for both HIV testing and diagnosis. A single proportion formula was employed to determine the sample size where P value is 0.05. With margin of error of 5% and non-response rate of 10%, the sample size needed was calculated to be 427 clients.

The two dependent variables used to measure the opportunity lost were missed opportunities for HIV test and missed opportunities for HIV diagnosis. The independent variables were: Age, marital status, occupation, personal risk perception, previous test conditions, clients' service need.

Any clients attending selected health facilities age above 13 years and below 64 years were used as inclusion criteria for participating in the study. This age range was based on CDC recommendation for routine HIV testing. However, HIV positive clients who were already diagnosed and were on treatment, clients coming for VCT service, patients who were not in clear mental state due to different health condition, patients who cannot stay interview time for the need of urgent medical attention were excluded from the study.

The two important operational definitions used during the study were 'missed opportunities for HIV test and missed opportunities for HIV diagnoses'. The first definition was used for clients who came to the health institution seeking different services and failed to be initiated by the service providers for HIV testing. While the later was used for the proportion of clients who were found to be HIV positive but failed to be diagnosed by service providers.

In order to assess the degree of lost in opportunities for HIV testing, operationalzing the missed opportunity for HIV test variable was applied:Level-1 missed opportunities for HIV test was meant to clients who were not initiated for HIV test by service providers after coming to health facilities for any service. The second level of missed opportunities for HIV testing was used for those clients who were not initiated for HIV test by service providers and latter become willing to have the test at the time of interview after initiated by data collecting counselors trained on HCT. The third level of missed opportunities for HIV test was operational for clients who were not initiated by service providers but when initiated by data collecting counselors, they accepted the test. The fourth level of missed opportunities for HIV test, the severe degree of the loss, is for clients who were initiated by data collecting counselors trained on HCT, offered the test and post-test result were positive.

Data collection in this study made use of exit interview of clients coming to health facilities seeking different health care services. The interview questions included clients' reasons for facility visits, clients' response on whether the providers had asked them on their previous test for HIV or not, their willingness to have HIV test, clients ever initiation by health workers to have an HIV test in any of their visits in the past one year, clients degree of self risk perception for HIV infection, clients ever test for HIV test, their current willingness to have the test, clients' readiness to have the test at the time of interview and if they became ready for initiation; performing of HIV test and providing post test counseling. At the end, clients' charts were traced and what was documented by the service providers reviewed and analyzed. The data collectors were licensed trained nurses on Provider Initiated Testing and Counseling (PITC). The total sample size (427 clients) were allocated to each health facilities based on the proportion of average clients served each day after taking the previous three months average daily clients load. Every third clients were interviewed using systematic sampling from each health facilities till the allocated proportion reached. Charts of clients who were in the exclusion criteria and who refused to participate were replaced by the subsequent chart of the third client. Service providers, supervisors and the principal investigator were kept blinded through unlinked anonymous method. This allowed the data collecting counselors only to link the information with the clients. The clients' charts were coded and later reviewed for detailed documented information.

To ensure a quality data collection, pretest was conducted in 10% of clients. And 5% of the collected data were assessed daily. In order to keep the quality of data clear from service providers' bias, service providers were kept blinded about the research area till the completion of data collection.

The data were entered and analyzed separately using SPSS version 15.0. Bivariate and multivariate logistic regression analysis was performed for comparison, association of results and rule out confounding factors among the variables. The over all study findings were compiled and presented using tables and statements.

Data collection was started after permissions were obtained from Research and Publication Office (RPO) and School of Public Health of the University of Gondar. Full consent statement was explained to each respondent in order to get permission based information. The confidentiality of the test results was kept using coded registrations and through unlinked blinding of both service providers and the principal investigators.

The result of this study is believed beneficiary for different disciplines working over HIV/AIDS areas including policy makers, planners, donors, program implementers, trainers, service providers and health professionals at different levels.

## Result

Of the total of 438 clients requested for interview, 427 clients (97.5% respondent rate) aged between 15 to 64 years were interviewed. The mean age of the study participants was 28.84 years with a standard deviation of ±10.29. Among all clients participated, 31.1% (133) were males and 68.9% (294) were females. (Table 1)

**Table 1:** Socio demographic characteristics of clients treated at Dessie health facilities, North East Ethiopia, December/2008(N = 427)

Variable	Frequency	Percent
**Sex**		
Male	133	31.1
Female	294	68.9
**Age**		
15-24 Yrs	177	41.5
25-34 yrs	142	33.3
35-44 yrs	65	15.2
45-54 yrs	28	6.5
55-64 yrs	15	3.5
**Address**		
Dessie town	267	62.5
Other woredas	150	37.5
**Marital status**		
Not married	140	32.8
Married	239	56.0
Divorced	31	7.2
Widowed	17	4.0
**Educational status**		
No formal education	75	17.6
1-4 class	52	12.2
5-8 class	91	21.3
9-10 class	90	21.1
11-12 class	61	14.3
>12 class	58	13.6
**Religion**		
Orthodox	170	39.8
Muslim	244	57.1
Catholic	3	0.7
Protestant	10	2.4
**Occupation**		
Student	82	19.2
Unemployed	22	5.2
House wife	141	33.0
Government employee	64	15.0
Private work	118	17.7
**Monthly income**		
<= 200 Birr	27	6.3
201-500 Birr	88	20.6
501-1500 Birr	146	34.2
>= 1500 Birr	38	8.9
I don't know	128	30.0

The majority, 34% (145/427), of clients' reason for visit was for medical treatment. Clients demanding family planning and gynecological care comprised 29.6% (126/427) of the visitors. Clients who needed emergency treatment were 13.3% (57/427) and who came for follow-up were 9.1% (39/427). Clients who came for ANC were 9.1% (39/427).

From all clients who came to the health facilities, only 24.4% (104/427) were asked by service providers whether they had previously made HIV test or not. Moreover, the clients' risk for HIV infection was inquired by service providers for 23.2% (99/427) clients. Initiation for HIV test through questioning willingness of clients for HIV test was not conducted by service providers for 76.1% (325/427) of clients. Among 23.9% (102/427) clients who were asked for their willingness by service providers, 67.6% (69/102) were willing to have the HIV test. Among non-willing clients, the major reason, 56.2% (18/32), for not willing was that they have no risk or they know they would be HIV negative. The other important reason, 31.3% (10/32), was that they were not ready for the test and they wanted more time to discuss with their partners.

In order to assess the past lost opportunities for HIV testing, clients' previous one year visit to any health facilities was asked. Most clients 73.3% (313/427) visited health facilities at least once in the previous one year where they could get counseling and testing for HIV. During this time, the large segment of clients, 46.9% (147/313), visited the health facilities four times or above. From the total clients who at least visited health facilities once in the past one year, 75.2% (228/303) were not initiated for HIV testing. However, a good number of them, 22.1% (67/303), suspected themselves of HIV infection when they felt ill.

Levels of self risk perception of clients for HIV infection were investigated in order to explore clients' insights against their actual risk behaviors for HIV infection. Accordingly, the greater part of clients, 72.8% (311/427), perceived themselves as having no risk for HIV infection. The majority, 73.6% (229/311), thought so because they were living in one to one relationship with their partners while 4.2% (13/311) thought so because of their use of condom. Of the total clients, 23.7% (101/427) perceived themselves as having high risk or some risk for HIV infection.

When previous HIV test status of clients was assessed, a great part of clients, 42.2% (180/427), had never tested for HIV in their life and 29.0% (124/427) had only tested once. Out of all the tested clients, the majority, 50.2% (124/247), had only tested once. The greater part, 73.8% (180/244), of clients had made their tests within the last one year. The mean last HIV test duration was found to be 12.95 months with standard deviation of ±17.04 months. Among the clients who ever tested for HIV, only 28.7% (70/244) were tested through opt out approach. While the greater part, 88.1% (215/244), were tested through VCT approach. A large number of clients, 42.2% (95/225), who started sexual intercourse and ever tested for HIV had never tested with their partners. All clients were asked by interviewer for their willingness for HIV test at the time of interview and 76.3% (326/427) were willing to have HIV test. Among 326 clients who showed willingness for HIV test at the time of interview, 37.7% (123/326) were ready to have the HIV test on the same day. However, HIV test was actually offered at the time of interview for 91.1% (112/123) clients who accepted the test. The major, 54.4% (155/285), reason of clients who were not willing for HIV test at the time of interview was either they were not ready or they wanted to discuss with their partner.

Among the clients tested for HIV by data collecting trained counselors at the time of interview, 86.6% (97/112) were HIV negatives while 13.4% (15/112) were HIV positives. Of HIV positive clients, 40.0% (6/15) were not willing to disclose their results to their partners. Moreover, half of the clients, 50.0% (3/6), who were not willing to tell to their partners were also refused to disclose their results to no other person.

**Figure 1:**
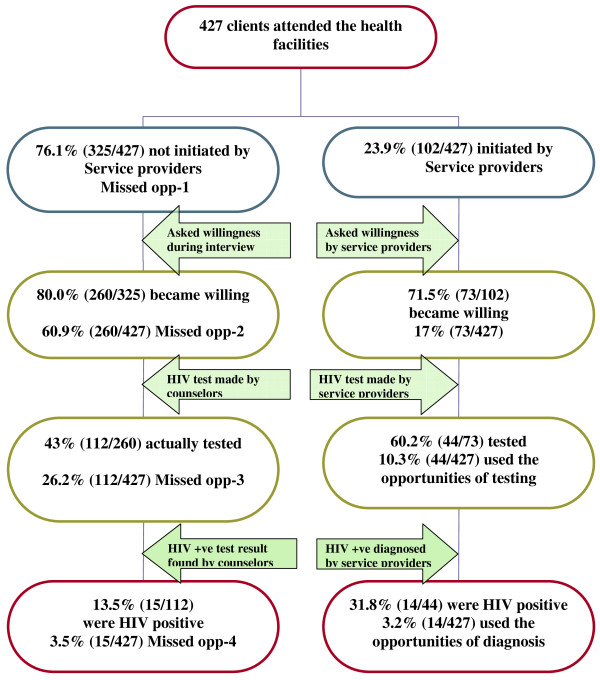
**Schematic drawing showing the proportion of missed opportunities for HIV testing in different levels**.

Investigating the missed opportunities for earlier HIV testing and diagnosis were the main objectives of why this study was conducted. (Figure 1). Among all clients coming to health facilities, the majority, 76.1% (325/427), missed the opportunities for HIV testing level 1. Of all not initiated clients by service providers, 80.0% (260/325) became willing when initiated by interviewers. The majority, 60.9% (260/427), of clients who were not initiated by service provider were willing to have HIV test when initiated by interviewers; which was the second level of missed opportunities for HIV testing. Among clients who became willing, 43.0% (112/260) actually tested for HIV. This made the third level of missed opportunities for HIV testing 26.2% (112/427). The sever degree of lost opportunities for earlier HIV testing occurred for 13.4% (15/112) of the tested clients; when clients failed to be initiated by service provider, but later initiated by interviewer, tested and found to be HIV positive. The fourth level of missed opportunities for earlier HIV testing occurred in 3.5% (15/427) of clients coming to the health facilities. Out of all clients, health professionals were able to diagnose 3.2% (14/427) HIV positive clients missing the diagnosis of 3.5% (15/427). As a result, the greater part of HIV positive clients, 51.7% (15/29), lost the opportunities of being diagnosed their sero status by service providers. (Table 2)

**Table 2:** Missed opportunities for earlier HIV testing and diagnosis of clients treated at Dessie health facilities, North East Ethiopia, Dec 2008(N = 427)

Variable	Frequency	Percent
**Missed opportunities for HIV testing level 1**		
Yes	325	76.1
No	102	23.9
**Missed opportunities for HIV testing level 2**		
Yes	260	60.9
No	167	39.1
**Missed opportunities for HIV testing level 3**		
Yes	112	26.2
No	315	73.8
**Missed opportunities for HIV testing level 4**		
Yes	15	3.5
No	412	96.5
**Tested by service provider (N = 44)**		
Negative	30	68.2
Positive	14	31.8
**Test acceptance rate after initiated by service providers (N = 73)**		
Offered	44	60.3
Not offered	29	39.7
**Tested during interviewing (N = 112)**		
Negative	97	86.6
Positive	15	13.4
**Test acceptance rate after initiated by interviewer only**		
Offered	112	26.2
Not offered	315	73.8
**Tested clients by service provider and by interviewer (N = 156)**		
By interviewer	44	28.2
By service provider	112	71.8
**Overall test acceptance rate by service providers and interviewers**		
Offered	156	36.5
Not offered	271	63.5
**Missed opportunities for HIV diagnosis (N = 29)**		
HIV positive result after tested by interviewers	15	51.7
HIV positive result after tested by service providers	14	48.3

Note:

**Missed opportunities for HIV testing level 1**-Clients who were not initiated for HIV test by service providers after coming to health facilities for any service.

**Missed opportunities for HIV testing level 2**-Clients who were not initiated for HIV test by service providers and latter became willing to have the test by data collecting counselors.

**Missed opportunities for HIV testing level 3**-Clients who were not initiated by service providers but when initiated by data collecting counselors, they accepted the test.

**Missed opportunities for HIV testing level 4**- clients who were initiated by data collecting counselors, offered the test and post-test result became positive.

**Missed opportunities for HIV diagnosis**- The proportion of clients who were failed to be diagnosed by service providers and later found to be HIV positive after tested by data collecting counselors.

The association of missed opportunities for HIV testing level 1 with socio demographic variables was assessed using bi-variate and multivariate analysis. Accordingly, sex, marital status, and religion of clients had no association with the lost opportunities for HIV testing. Family monthly income had association with the lost opportunities for HIV test in bivariate analysis, whereas educational status and occupation of clients had association in both bivariate and multivariate analysis. Individuals with educational status between 11-12 grade lost the opportunities for HIV testing 3.02 times more likely compared to the clients with no formal education. OR 3.02(1.130-8.084) (P = 0.028). However, government employee had 0.227 times less likely of missing the opportunities for HIV testing compared to the students. OR 0.227(.064 - .806) (P = .022) (Table 3)

**Table 3:** Missed opportunities for HIV testing vs. socio economic characteristics of clients attending health facilities at Dessie town, North East Ethiopia, December 2008

	Missed Opportunities for HIV testing level 1			
			
Variable	Yes	No	Crude OR(95%CI)	Adjusted OR(95%CI)
**Sex**				
Male	98	35	1	
Female	227	67	.83(.52 - 1.32)	.56(.29-1.06)
**Marital status**				
Single	109	31	1	
Married	181	58	3.52(.21-57.84)	4.05(.12-143.16)
Divorced	26	5	3.12 (.19-50.68)	4.48(.13-52.18)
Widowed	8	7	5.20 (.28-97.62)	4.20(.11-167.87)
**Educational status**				
No formal Educ.	61	14	1	
1-4 class	44	8	2.86(1.31-6.27)*	1.74(.53-5.68)
5-8 class	67	24	3.61(1.44-9.06)*	2.36(.69-8.02)
9-10 class	76	16	1.84(.91-3.71)*	1.49(.56-3.97)
11-12 class	42	19	3.56(1.64-7.75)*	3.02(1.13-8.08)*
>12 class	34	23	1.45(.68-3.09)	1.33(.54-3.25)
**Occupation of client**				
Student	64	18	1	
Unemployed	19	3	.43(.13-1.38)	.74(.18-3.09)
House wife	101	4	.76(.15 -3.80)	.58(.10-3.26)
Government employee	42	22	.31(.10-.92)*	.22(.06-.81)*
Private work	66	15	.23(.07-.74)*	.61(.15-2.42)
Other	33	4	.53(.16-1.73)	.51(.14-1.90)
**Monthly income in Birr**				
<= 200 Birr	20	7	1	
201-500 Birr	76	12	.69(.26-1.82)	.74(.26-2.13)
501-1500 Birr	102	44	1.53(.72-3.25)	1.85(.78-4.37)
>= 1500 Birr	24	14	.56(.32-.98)*	.76(.39-1.49)
I don't know	103	25	41(.19-.92)*	.63(.24-1.64)

## Discussion

This study identified the extent to which missed opportunities for earlier HIV testing and diagnosis occurred among clients who came to health facilities seeking different health services. The study showed the majority of clients, 76.1% (325), visited the health facilities were not initiated for HIV testing and returned home losing the opportunities of having HIV testing and diagnosis. Of these clients 20.9% (68) perceived themselves to be at some or high risk for HIV infection. The majority of not initiated clients 74.2% (241) had previously sought health care at least once at site where HIV counseling and testing is commonly offered in the past one year. Of those clients who sought health care in the past one year, 87.1% (210) were also previously lost the opportunities of initiation for HIV test in their past visits to the clinic. This fact indicates that missed opportunities for HIV testing were very high and were occurring repeatedly on the same clients within short period of time.

When tested for HIV, 13.4% (15) clients who actually accepted the test were HIV positive. Of HIV positive clients, 6/15 felt themselves to be at some or high risk while 60% (9) felt themselves as having no risk for HIV infection demonstrating the problem of missing the majority of HIV positive patients if we test only clients who perceive themselves as high risk. The majority, 86.7% (13), of HIV positive patients had previously sought care at a site where HIV testing was commonly offered or at a site where risk could have been assessed and HIV counseling and testing offered. This fact indicates most of HIV positive result could have been diagnosed earlier or infection could have been averted if initiation and testing for HIV had been done prior to their infection.

This study finding is consistent with the study conducted at a South Bronx, New York which showed that of the 807 patients enrolled, 411 (51%) had never been offered HIV CT previously. Of these patients, 50% had sought care at a site where HIV CT is commonly offered, 15 (4%) of the 411 patients were HIV positive [13]. The finding from our study demonstrated a higher missed opportunities loss for earlier HIV testing and a high positivity rate when compared to the study conducted at South Bronx. This could be due to low priority given by service providers for HIV testing and due to the high HIV prevalence rate in our community. This study finding substantiates the problem of risk based HIV testing initiation as the majority, 60%, of HIV positive patients felt themselves no risk for HIV infection. Thus, the study finding supports the concept which states 'in settings serving clients at increased behavioral and clinical risk for HIV infection, targeting HIV testing based on reported risk factors misses many HIV-infected clients'[14].

The study also verified the high acceptance rate for HIV test among clients offered initiation for the test; 76.3% (326) clients were willing to have the test. The overall test acceptance rate after clients were initiated both by service providers and VCT trained data collecting counselors were 36.5% (154). However, the test acceptance rate varies among service providers and data collecting interviewer with the former being 60.9% (44) and the later being 26.9% (112). The positive result rate for test conducted by service provider was 31.8% (14) and for data collecting counselors was 13.4% (15). The high test positivity rate and high test acceptance rate result by service providers revealed the use of HIV test by service providers as diagnostic rather than as screening approach. The overall test acceptance rate and positivity rate is very high when compared to the recommendation which states 'in the case of a screening test, a positive result of 1 in 20 or 1 in 100 would be perfectly acceptable' [10].

The willingness and test acceptability rate of this study is similar to a study conducted at Arbaminch Hospital, southern Ethiopia, to assess the acceptability of HIV counseling and testing among TB patients under routine care conditions. This study showed 73% willingness to be tested and 58% of those willing accepted the test with the overall acceptability rate of 35% and 20.6% HIV positive result [7]. The study conducted in Addis Ababa on clients who showed signs and symptoms of HIV infection revealed a pre-test and post-test acceptability rates of 0.98 and 0.96 respectively and the overall acceptability rate of all study participants of 0.67[22]. The lower test acceptance rate of our study in comparison with this study might be because of the involvement of large clients who didn't presented with HIV sign and symptoms.

The major reasons, 54.4% (155), given by clients who were not willing for HIV testing at the time of interview were either they were not ready or they wanted to discuss with their partner. This finding partially supports the stated fact which says 'the main reasons for avoiding or delaying testing were fear of learning one is HIV-positive, thinking one was unlikely to have been exposed to HIV, thinking one was HIV negative, not wanting to think about the possibility of being HIV-positive, and thinking there is little that can be done about being HIV-positive' [5].

The findings from the assessment of missed opportunities for HIV diagnosis by service providers were unacceptably high, 51.7% (15). This fact indicates, should initiation for HIV testing were not done by data collecting counselors,15 clients could have been failed to be diagnosed as HIV positive and could have lost the subsequent work-up for referral and the involvement in prevention strategies. The major contributory factors for this degree of loss in opportunities for HIV diagnosis seems the high missed opportunities for HIV testing occurred at the facilities. This finding is consistent with the study finding conducted in developed countries set ups at Brighton and Sussex University Hospitals in UK, which showed in almost half (48%) of the symptomatic individuals that presented to health care, the diagnosis of primary HIV infection was not made[23].

Moreover, those clients coming with primary HIV infection were not included in this study as the antibody test can't diagnose HIV at primary infection time. Therefore, clients with primary HIV infection were either not tested (not willing to have the test) or remained undiagnosed after the test was conducted through this study. If diagnosis of primary HIV infection was possible to be made in this study, it could have made the missed HIV diagnosis more than result the found, 51.7%.

Large number of clients, 42.4% (180), had never tested for HIV in their life time which capitalizes the importance of reaching this sum of people using the opportunities of their coming to the health facilities. The greater part, 71.1% (128) of the never tested participants, attended health facilities where HIV test could be conducted in the past one year. This indicates that the never tested clients have lost opportunities for HIV test in near past and shows how much repeatedly losing the opportunities for HIV at health facilities contributes for clients to remain untested for HIV. Most of clients, 82.4% (149), who never tested for HIV were not initiated for HIV testing by service providers. A large number, 19.4% (12), of clients who never tested for HIV were found to be HIV positive when tested. The findings showed clients who never tested for HIV constituted the larger part, 80% (12), of HIV positive cases detected during interview. This indicates that clients who never tested for HIV were at greater disadvantage of losing the opportunities for HIV testing and diagnosis than those who has at least a test in their life time. This pools of positive cases among the never tested clients showed that there could be much more detection of HIV positive cases if more effort is made to enroll clients who never tested to undergo HIV test.

The finding also revealed individuals with educational status at tertiary grade to loss the opportunities for HIV testing 3.02 times more likely compared to the clients with no formal education. This may be due to less emphasis made by service providers on the educated people to initiate for HIV test thinking that they can protect themselves. However, government employees had 0.227 times less likely of missing opportunities for HIV testing compared to the students. This could be due to the less emphasis given by service providers for HIV test initiation of students thinking that they are less vulnerable to HIV infection when compared to the employed clients.

The study showed the degree of opportunities lost on HIV testing and diagnosis and can be used as a baseline for interventions to be carried out in HIV preventive areas. As strength, the study design included the testing of those clients who were willing and accepted the HIV initiation by trained data collecting counselors. This helped out to assess the degree of missed opportunities for HIV testing in different levels and to visualize the real severity of the problems. As a limitation, missed opportunities for HIV testing and diagnosis at the private sector was not assessed. For clients tested for HIV, if there were clients infected with HIV but in window period, diagnosis of HIV infection using serological rapid test was not possible. This could have raised the proportion of missed opportunities for HIV testing and diagnosis higher than what is found in this study.

## Conclusions

This study demonstrated that the large segment of clients who came to health facilities was not ever tested for HIV. The finding also verified that there were unacceptably high and repetitive missed opportunities for earlier HIV testing and diagnosis occurring at health facilities. Targeting HIV test initiation based on reported risk factors misses many HIV-infected clients. The overall HIV test acceptability rate of the initiated clients was found to be high. As a result, routine requesting of clients' willingness for HIV testing should be conducted by all service providers irrespective of clients' risk behaviors for HIV infection or the type of service they need.

## Competing interests

The authors declare that they have no competing interests.

## Authors' contributions

NWF: Drafted the manuscript, designed and conducted the study, performed the statistical analysis and carried out the manuscript write-up. ADF: Commented on the manuscript draft, advised while designing and conducting the study, helped the statistical analysis and in re-writing the final document. Both authors read and approved the final manuscript.

## Pre-publication history

The pre-publication history for this paper can be accessed here:

http://www.biomedcentral.com/1471-2458/10/362/prepub
